# Neurological and behavioral features of locomotor imagery in the blind

**DOI:** 10.1007/s11682-020-00275-w

**Published:** 2020-04-02

**Authors:** Kaoru Amemiya, Tomoyo Morita, Satoshi Hirose, Tsuyoshi Ikegami, Masaya Hirashima, Eiichi Naito

**Affiliations:** 1 Center for Information and Neural Networks (CiNet), National Institute of Information and Communications Technology (NICT), 1–4 Yamadaoka, Suita, Osaka, 565-0871 Japan; 2 Institute for Open and Transdisciplinary Research Initiatives, Osaka University, 1-1 Yamadaoka, Suita, Osaka, 565-0871 Japan; 3 Graduate School of Frontier Biosciences, Osaka University, 1-3 Yamadaoka, Suita, Osaka, 565-0871 Japan

**Keywords:** Blind, Functional magnetic resonance imaging, Motor imagery, Supplementary motor area, Cerebellum

## Abstract

In people with normal sight, mental simulation (motor imagery) of an experienced action involves a multisensory (especially kinesthetic and visual) emulation process associated with the action. Here, we examined how long-term blindness influences sensory experience during motor imagery and its neuronal correlates by comparing data obtained from blind and sighted people. We scanned brain activity with functional magnetic resonance imaging (fMRI) while 16 sighted and 14 blind male volunteers imagined either walking or jogging around a circle of 2 m radius. In the training before fMRI, they performed these actions with their eyes closed. During scanning, we explicitly instructed the blindfolded participants to generate kinesthetic motor imagery. After the experimental run, they rated the degree to which their motor imagery became kinesthetic or spatio-visual. The imagery of blind people was more kinesthetic as per instructions, while that of the sighted group became more spatio-visual. The imagery of both groups commonly activated bilateral frontoparietal cortices including supplementary motor areas (SMA). Despite the lack of group differences in degree of brain activation, we observed stronger functional connectivity between the SMA and cerebellum in the blind group compared to that in the sighted group. To conclude, long-term blindness likely changes sensory emulation during motor imagery to a more kinesthetic mode, which may be associated with stronger functional coupling in kinesthetic brain networks compared with that in sighted people. This study adds valuable knowledge on motor cognition and mental imagery processes in the blind.

**Electronic supplementary material:**

The online version of this article (10.1007/s11682-020-00275-w) contains supplementary material, which is available to authorized users.

## Introduction

Motor imagery is a multisensory emulation process associated with an action, especially when people imagine a previously experienced action. From this sensory perspective of motor imagery, the imagery likely contains components of multisensory (kinesthetic, visual, auditory, and vestibular) experiences. These are a substitute for the sensory feedback that would normally arise in association with an overt action, probably by accessing memories of the experienced action (Annett 1995, 1996; Grush 2004; Guillot et al. 2009; Hanakawa 2016; Naito et al. 2002).

Among these possible sensory modalities, kinesthesia and vision have been considered as particularly important sensory components in motor imagery (Imbiriba et al. 2006; Munzert et al. 2009). Owing to the multisensory nature of motor imagery, even when participants are instructed to generate kinesthetic motor imagery (imagining their own actions as if they actually move their body parts and feel sensations of movement from the first-person perspective; Jackson et al. 2006), visual components often infiltrate motor imagery (Hétu et al. 2013; Munzert et al. 2009). However, this holds true only for participants with normal sight whose sensory information processing largely relies on vision in daily life. Yet, little is known of how blind participants who have poor long-term visual experience emulate sensory experiences during motor imagery. In the present study, we examined how long-term blindness influenced sensory experience during motor imagery and its neuronal correlates by comparing the data obtained from blind and sighted participants.

In sighted participants, brain networks involving kinesthetic (first-person perspective) and visual (third-person perspective) modes of motor imagery differed when imagining hand actions (Guillot et al. 2009; Ruby and Decety 2001; Sirigu and Duhamel 2001; Solodkin et al. 2004; Stinear et al. 2006) and entire-body actions (Olsson et al. 2008), in addition to commonalities in the involved brain structures (Hétu et al. 2013; Solodkin et al. 2004; Fourkas et al. 2006a; Fourkas et al. 2006b). Viewed collectively, the involvement of visual cortices in visual motor imagery (Guillot et al. 2009; Olsson et al. 2008; Solodkin et al. 2004) and cerebellum in kinesthetic motor imagery (Guillot et al. 2009; Naito et al. 2002; Olsson et al. 2008) has been consistently reported. In addition, the supplementary motor area (SMA), premotor area (PM), inferior frontal cortices (IFC), and superior parietal lobule (SPL) are reported to show modality-non-specific involvement during motor imagery (Hétu et al. 2013; Ruby and Decety 2001; Solodkin et al. 2004), though the SMA and PM may show preference for kinesthetic motor imagery (Guillot et al. 2009; Naito et al. 2002; Olsson et al. 2008).

Early and late blind people may use distinct motor representations to those of sighted people during motor imagery (Imbiriba et al. 2006; Imbiriba et al. 2010; Imbiriba et al. 2013). Motor imagery of blind (especially congenitally blind) people is likely performed from a first-person (egocentric) perspective using a kinesthetic mode (Imbiriba et al. 2010). If this view is correct, the cerebellum and visual cortices should be more strongly involved in motor imagery of blind and sighted people, respectively, owing to the visual-dominant information processing style in the latter. In addition to group-specific neural substrates, group-non-specific (consistent across groups) involvement of the secondary motor and frontoparietal areas is expected.

There is a limited number of functional magnetic resonance imaging (fMRI) studies addressing the neural substrates underpinning motor imagery in blind people (Deutschländer et al. 2009a, b; Jahn et al. 2009). These studies used a locomotor imagery task and showed that imagery in blind participants more strongly activated multisensory vestibular areas and primary sensorimotor cortices (Deutschländer et al. 2009a, b), whereas that of sighted participants activated the parahippocampal regions and deactivated multisensory vestibular areas (Deutschländer et al. 2009a, b; Jahn et al. 2004; Jahn et al. 2008; Jahn et al. 2009).

A meta-analysis in sighted people revealed that gait motor imagery activated the secondary motor and frontoparietal regions, but no parahippocampal activation was observed (Hétu et al. 2013). Therefore, it is possible that the parahippocampal activation is task-specific and is associated with imagination of locomotor spatial navigation and the visual environment in a long basement floor during locomotor imagery, which was visually experienced in prescanning training (see above references). Hence, if sighted participants do not have such visual experiences during prescanning training, such activation should not emerge during locomotor imagery during scanning. Conversely, vestibular and sensorimotor activation during locomotor imagery of blind participants implies greater reliance on vestibular and somatic information (Deutschländer et al. 2009a, b).

Hence, in the present study, we adopted a different type of locomotor task of either walking or jogging around a circle. If the aforementioned brain activation/deactivation patterns is generalizable to locomotor imagery, the same patterns of brain activation/deactivation would be expected in the present task.

In our training before fMRI scanning, both blind and sighted participants first performed either walking or jogging around a circle with their eyes closed, and subsequently imagined each action. To assess if an imagined movement obeyed the same motor rules as those of a real movement (Decety et al. 1989; Decety and Jeannerod 1995; Jeannerod 1994; Papaxanthis et al. 2002; Parsons 1987; Sirigu et al. 1996), we evaluated the relationship between actual time required to complete walking or jogging along the circle and imagery time required to complete each imaginary action in training. We expected that imagery time would approximate actual time regardless of walking or jogging, and that walking imagery time would be longer than jogging imagery time.

In the fMRI experiment, we scanned brain activity while participants imagined the experienced locomotor actions with their eyes closed. We explicitly instructed them to generate kinesthetic motor imagery from the first-person perspective. After each fMRI run, we instructed them to rate the degree to which their imagery became kinesthetic or spatio-visual. In this subjective rating, we examined how sightedness and blindness influenced subjective multisensory experiences during motor imagery. We expected that even when participants were instructed to generate kinesthetic motor imagery, visual components would easily infiltrate motor imagery in sighted participants (see above), while blind participants would have relatively pure kinesthetic motor imagery.

In the fMRI data analysis, we first conducted conventional contrast analysis to assess group differences and between-group commonalities in imagery-related brain activation/deactivation. Here, we hypothesized consistent involvement of the secondary motor and frontoparietal areas between groups. In addition, we examined similarities and differences in brain activation/deactivation patterns between the present and previous studies (see above), especially for parahippocampal involvement in sighted participants, and vestibular and sensorimotor areas in blind participants, using region-of-interest (ROI) analysis. Next, we performed functional connectivity analysis to identify group-specific covariate patterns of activity between brain regions during the imagery task, which may be missed by contrast analysis (Morita et al. 2014; Zaki et al. 2007). Here, we expected group-specific involvement of visual cortices and the cerebellum in imagery of sighted and blind participants, respectively.

## Methods

### Participants

Fourteen blind (mean age, 32.4 ± 7.1 years, ranging from 22 to 42 years) and 16 age-matched sighted (mean age, 30.2 ± 5.1 years, ranging from 23 to 42 years) volunteers participated in the study. All participants were male and were healthy volunteers with no history of psychiatric disorders. In our recruitment of blind participants, we contacted the Japanese Blind Football Association. Thus, our blind participants included six blind soccer players (mean age, 31.7 ± 5.8 years; soccer experience more than 2 years) and eight age-matched (mean age, 33.3 ± 7.7 years) blind persons with no intensive soccer-playing experience. To match this participant composition for the sighted group, we recruited eight soccer players (mean age, 32.4 ± 7.1 years; soccer experience more than 9 years) and eight persons with no intensive soccer-playing experience. Participants’ handedness was confirmed using the Edinburgh Handedness Inventory (Oldfield 1971). All of the sighted participants and 10 of 14 blind participants were right-handers. In the blind group, three were ambidexters, and one was a left-hander.

Clinical features of blind participants are summarized in Table 1. The blind participants included three congenital blind, two early blind (blind onset ≤5 years), and nine late blind participants. Blindness was assessed by self-reports of each participant. The congenital and early blind participants reported difficulties with visual imagery (Table 1) but reported the ability for spatial imagery as a substitute for visual imagery (Arditi et al. 1988; Kaski 2002). They reported the ability to sense and imagine movements of another in the extrapersonal (unreachable) space and movements of their body parts not merely by kinesthesia but also by other multisensory cues (auditory, air flow, etc). All of the late blind participants reported the ability for visual imagery of their body movements. Based on this, we described visual imagery as spatio-visual imagery in the present study.

**Table 1:** Blind participants: summary of clinical features

	Age (y)	Sex	Handedne ss score*	Vision before blindness	Onset of total blindness (y)	Light sensitiviy at present	Cause of blindess	Visual imagery
sub1	29	M	6	Weak light sensitivity until 6 y	0	None	Unknown	No
sub2	32	M	89	Almost none	0	None	Cataracts	No
sub3	27	M	89	No visus after birth	0	None	Unknown	No
sub4	39	M	76	Almost none	1	None	Pediatric cancer in optic nerve	No
sub5	22	M	100	Congenital amblyopia, light sensitivity until 5 y	5	None	Retinal detachment	No
sub6	42	M	100	Normal vision	8	None	Retinal detachment	Yes
sub7	39	M	79	Normal vision	14	Yes	Congenital chorioretinal atrophy	Yes
sub8	42	M	100	Normal vision, eyesight reduction after ED	15	None	Congenital coats disease + Retinal detachment	Yes
sub9	39	M	100	Normal vision	17	Occasional	Uveitis + Glaucoma	Yes
sub10	30	M	20	Congenital amblyopia (visus = 0.04), eyesight reduction after ED	18	Yes	Microphthalmia + Coloboma	Yes
sub11	38	M	-6	Normal vision until 10 y, eyesight reduction after 10 y	20	Yes	Retinitis Pigmentosa	Yes
sub12	26	M	67	Normal vision until 6 y, Visus = 0.06 after 7 y	23	Yes	Uveitis	Yes
sub13	41	M	100	Normal vision, eyesight reduction after ED	23	None	Congenital glaucoma	Yes
sub14	41	M	−50	Visus = 0.02 with light sensitivity	27	Almost none	Congenital glaucoma + Retinal detachment	Yes

*Handedness determined according to short version of Edingburgh test. ED = early adolescence

We first evaluated motor imagery in each participant using a controllability of motor imagery (CMI) test (Naito 1994; Nishida et al. 1986), a unique and reliable measure that evaluates an individual’s ability to generate, manipulate, and hold motor imagery from a first-person perspective (see more in Supplementary Material). Individuals who are skilled at having kinesthetic motor imagery score higher in the CMI test (Naito et al. 2002). The average CMI score across participants was 44.8 (ranging from 35 to 53) and 41.7 (ranging from 24 to 54) for the sighted and blind groups, respectively. No difference was observed between groups (*df* = 28, *t* = 0.8, two-sample t-test, *p* = 0.26), indicating similar between-group CMI.

The protocol used for this study was approved by the ethics committee of the National Institute of Information and Communications Technology. We explained the details of the study to the participants before the start of the experiment. All participants provided written informed consent. In cases where blind participants could not provide their signatures, we obtained their oral informed consent and written informed consent signed by an experimenter or their guardians in their presence. The experiment was conducted in accordance with the principles of the Declaration of Helsinki (1975).

### Training

Before fMRI experiments, all participants experienced blind walking and jogging around a circle of 2 m radius in a gymnasium. In a previous study, participants experienced straight running and walking on a basement floor, which was long enough to walk or run in a straight line towards one direction for more than 20 s (Deutschländer et al. 2009a, b; Jahn et al. 2009). In the present study, we adopted the circular locomotion task, which may require an evaluation of the trajectory radius, walked distance, head-turning angle, and body tilt angle. The circular trajectory should be generated from spatial and motor memory without vision. The geometrical properties of the circle must be translated into appropriate locomotor patterns using inertial and somatic signals (Takei et al. 1997). Thus, performing this task requires vestibular and somatic information.

Before training, all participants wore a pair of eye patches and eye mask in the preparation room and then entered the gym, precluding visual information about the experimental environment in the gym. During training, participants were presented with white noise through a wearable headphone. Action-associated visual and auditory experience was completely eliminated in all participants. These settings helped to equalize potential sensory experience during training between groups.

The training comprised four conditions: clockwise walking, counter-clockwise walking, clockwise jogging, and counter-clockwise jogging, comprising three trials each. We employed both clockwise and counter-clockwise conditions to minimize possible bias due to individual preference of any particular direction. The walking condition was performed first, followed by the jogging condition. The direction was pseudo-randomized across participants in each condition. Before each trial, an experimenter (TI) guided them to walk (or jog) around the circle from the start position to the end position (start and end positions were the same). The experimenter walked (or jogged) alongside participants by holding their externally facing hand. After completion of this guided trial, participants started an unguided trial. The pair of guided and unguided trials was repeated three times in each condition. Before commencing training, instructions were provided by removing their headphones. Participants were instructed to walk (or jog) along the circle as accurately as possible, and to stop walking (or jogging) and to say ‘I’m finished’ when they believed they had returned to the start position. No post-trial performance feedback was provided.

Walking and jogging trajectories were recorded using a motion capture system (MAC3D system, nac Image Technology Inc.). Three-dimensional positions of three reflective markers attached on the head were recorded by 20 cameras at 200 Hz. The participants were able to create circle-like trajectories although many of them were imperfect circles. Based on the recorded movements, we calculated the time required to complete each action (from the initiation to termination of each action) in each trial, defined as actual time. In each trial, we calculated the path length traveled by summing up traveled distance between the initiation and termination of each action. We then calculated mean velocity of each action by dividing the path length with the actual time.

After completion of training, participants were asked to mentally rehearse (imagine) these actions, for both directions and each condition separately, as precisely as possible while seated. The order of the conditions was identical to the actual training. Each participant completed two imagery trials in each condition. They were instructed to say ‘start’ when they started imagining each action and ‘stop’ when they had completed the imaginary action. Duration was measured with a stopwatch and defined as imagery time.

We calculated mean actual time and mean imagery time for walking and jogging separately by pooling the data obtained from clockwise and counter-clockwise cases in each condition for each participant. In the analysis of actual and imagery times, we excluded the data obtained from two (one early and one late) blind participants, as walking trajectories of one participant were framed out from our recording space covered by the motion capture system and another participant showed outlier values (>3× standard deviation from the mean) in actual time.

For statistical evaluation of actual and imagery times, we first performed a three-way analysis of variance (ANOVA) that included one between-subject factor (Group [2]: sighted, blind) and two within-subject factors (Condition [2]: walking, jogging; Task [2]: actual, imagery). As we found a significant interaction between these three factors, we subsequently performed two-way ANOVAs. To evaluate actual and imagery times in walking and jogging conditions in each group, we first conducted a repeated measures ANOVA that included two within-subject factors (Condition [2]: walking, jogging; Task [2]: actual, imagery). Next, to evaluate possible group differences in actual and imagery times, we performed another ANOVA that included one between-subject factor (Group [2]: sighted, blind) and one within-subject factor (Task [2]: actual, imagery). The latter was performed separately for the walking and jogging conditions. As we observed a significant interaction between these two factors, we further performed two-sample t-tests with Bonferroni correction to evaluate possible group differences in actual time and imagery time separately in each condition.

We calculated mean velocity for walking and jogging separately by pooling the data obtained from clockwise and counter-clockwise cases in each condition for each participant. In this analysis, we were unable to calculate the mean velocity in the participant who was framed out from our recording space (see above). In the statistical evaluation of velocity, we performed a two-way ANOVA that included one between-subject factor (Group [2]: sighted, blind) and one within-subject factor (Condition [2]: walking, jogging). In this analysis, as we observed a significant interaction between these two factors, we further performed two-sample t-tests with Bonferroni correction to evaluate possible group difference in velocity in each. Corrected *p* values are reported in the results.

### fMRI experiment

The fMRI experiment was conducted on a separate day to the training day. Average days between the training and fMRI experiment were 14.0 ± 11.9 days. In the fMRI experiment, blindfolded participants were instructed to imagine either walking or jogging around the circle as if they actually performed the actions previously experienced on the training day. Before the fMRI experiment, they were explicitly instructed to imagine kinesthetic motor imagery of the experienced actions from the first-person perspective. We also informed participants that after each experimental run, they would be asked to rate how much they felt that their imagery was kinesthetic or spatio-visual. We explained to the participants that kinesthetic imagery meant imagining their own actions as if they actually moved their body parts and felt movement sensations, whereas spatio-visual imagery included imagining the actions as if they were observing the movements of someone else in the extrapersonal (unreachable) space, and also as if they saw their body parts moving from the first-person perspective during imaginary locomotion. For three congenital and two early blind participants who reported difficulties with visual imagery (Table 1), a slightly different phrase was used for explanation as follows: “spatio-visual imagery denotes spatial imagery of the actions as if you sense the motions of someone else in the extrapersonal (unreachable) space and also as if you sense your body parts moving during imaginary locomotion based on non-kinesthetic components (auditory, air flow, etc.)”. All participants reported that they understood these instructions.

Before they entered the MRI scanner, all participants (including blind participants) wore a pair of eye patches and an eye mask to eliminate any possible visual stimuli, which were kept on throughout the experiment. We started scanning their brains about 15–20 min after the visual deprivation. This was consistent across participants, as eye closure duration greatly affects activity in visual areas (Merabet et al. 2007; Weisser et al. 2005).

Participants subsequently lay in the MRI scanner. Their heads were immobilized with sponge cushions, and their ears were plugged. Both arms were naturally semipronated and extended in front of participants. Participants were told to relax their entire body without producing unnecessary movements and to think only of things relevant to the tasks assigned.

Each participant completed two experimental runs. Each run comprised eight imagery epochs, lasting 20 s each. The imagery epochs were separated by 10-s baseline periods. Each run also included a 20-s period before the start of the first epoch. In one epoch, participants were asked to imagine either clockwise or counter-clockwise and walking or jogging, resulting in four imagery conditions. Each condition was performed twice per experimental run in a pseudo-randomized order. During the experimental run, participants were given auditory instructions (e.g., clockwise, walk, start) through MR-compatible headphones to inform them of the action to be imagined and starting time. We also provided the instruction “stop” to notify the participants of the cessation time for each epoch. These instructions were generated by a computer. When participants thought they had completed one full circuit of imaginary walking or jogging around the imaginary circle within a 20-s imagery epoch, they were required to move on to the second circuit.

**Fig. 1:**
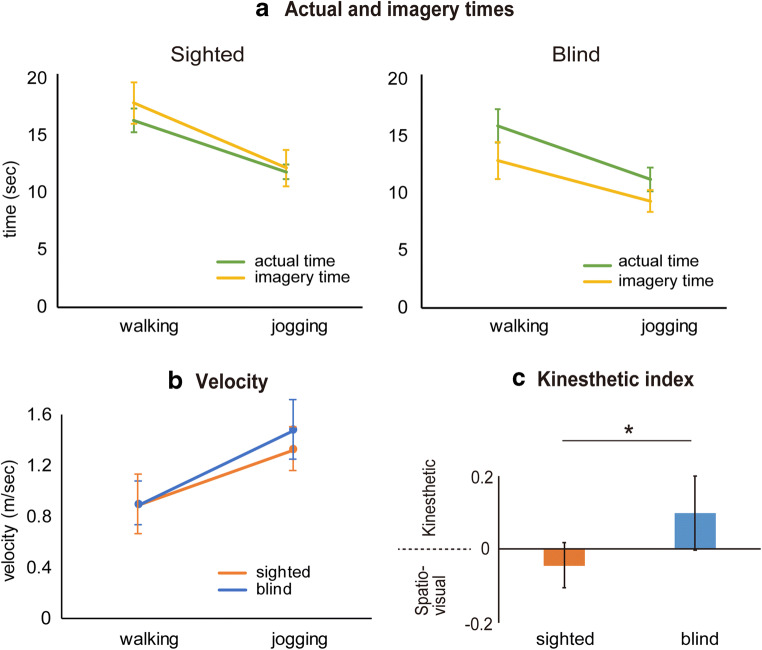
**a** Mean actual time (green) and mean imagery time (yellow) across participants for walking and jogging conditions in sighted (left) and blind (right) groups. In both groups, both actual and imagery times for walking were significantly longer than those for jogging. Only in the blind group, imagery time became significantly shorter than actual time regardless of walking or jogging. **b** Mean velocity across participants for actual walking and jogging conditions in sighted (orange) and blind (blue) groups. Walking velocity was the same between both groups, while jogging velocity was significantly faster in the blind group. **c** Result of psychological evaluation on motor imagery in each group. Participants rated to what extent their imagery became kinesthetic or spatio-visual. These scores were used to calculate the kinesthetic index. A kinesthetic index below 0 indicates higher spatio-visual score than kinesthetic score. The blind group (blue) reported significantly greater kinesthetic index value than that of the sighted group (orange). Asterisk indicates statistical significance (*p < 0.05). In all panels from a to c, error bars indicate standard deviation of the means across participants

After each experimental run, we asked the participants to rate the degree to which they felt that their imagery was kinesthetic or spatio-visual using a score from 1 (weak) to 5 (strong) for each component. We calculated kinesthetic index based on (kinesthetic score – spatio-visual score) / (kinesthetic score + spatio-visual score) for each participant to evaluate the extent to which their imagery became kinesthetic during multisensory motor imagery by excluding potential individual bias in scoring (i.e., a general tendency to score higher for both sensory aspects in some participants and lower in others). A kinesthetic index greater than 0 indicated that imagery was more kinesthetic; a value smaller than 0 indicated that imagery was more spatio-visual (Fig. 1c). Group means were calculated, and a two sample t-test was performed. To exclude the possibility that the data obtained from three congenital and two early blind participants affected the results, we also analyzed the data by excluding these data.

### fMRI data acquisition

Functional images were acquired using T2*-weighted gradient echo echo-planar imaging (EPI) sequences obtained using a 3.0-Tesla MRI machine (Trio Tim; SIEMENS; Germany) and a 32-channel array coil. We used a multiband imaging technique (multiband factor = 3). Each volume consisted of 51 slices across the entire brain acquired in an interleaved manner, with slice thickness of 3.0 mm. The time interval between two successive acquisitions from the same slice (TR) was 1000 milliseconds. Echo time (TE) was 27 milliseconds, and flip angle (FA) was 60°. The field of view (FOV) was 192 × 192 mm, and matrix size was 64 × 64. Voxel dimensions were 3 × 3 × 3 mm in the *x-*, *y-,* and *z*-axes. We collected 260 volumes in one experimental run.

### Imaging data analysis

#### Preprocessing

The first five volumes of each fMRI run were discarded because of unsteady magnetization. Imaging data were analyzed using Statistical Parametric Mapping (SPM8; Wellcome Trust Centre for Neuroimaging, London, UK) implemented in Matlab (Mathworks, Sherborn, MA). Initially, EPI images were realigned to the first image and then to the mean image. All participants had less than 3 mm of maximum (cut-off) motion in every plane (x, y, z) during the fMRI run. Thus, no data were excluded from the following analysis. The realigned images were normalized to the Montreal Neurological Institute (MNI) space (Evans et al. 1994). Finally, the spatially normalized functional images were filtered using a Gaussian kernel with a full-width-at-half-maximum (FWHM) of 8 mm in the *x-*, *y-,* and *z*-axes.

#### Analysis of imagery-related activation and deactivation in each group

After preprocessing, we evaluated imagery-related activation and deactivation using a general linear model (GLM) in each participant. The design matrix contained a boxcar function for the imagery epoch (task-related regressor), which was convolved with a canonical hemodynamic response function. We constructed appropriate images to examine brain regions showing imagery-related activation (imagery > baseline) and deactivation (baseline > imagery) in each participant. In these analyses, we pooled the data obtained from two (walking and jogging) conditions as previously reported (Deutschländer et al. 2009a; Jahn et al. 2008). The images from all participants were entered into a second-level random effects group analysis to accommodate inter-participant variability (Holmes and Friston 1998). A one-sample *t*-test was performed separately for each group. In second-level analyses, we first generated a voxel-cluster image using an uncorrected height threshold of *p* < 0.001 in each group. For statistical inference, we used an extent threshold of *p* < 0.05 at the cluster level after correction for multiple comparisons using the family-wise error rate (FWE) in the whole brain. We consistently used this conservative threshold in subsequent analyses, except for region-of-interest (ROI) analysis (see below). We referred to the cytoarchitectonic probability maps in the MNI standard brain of the SPM anatomy toolbox v2.2b (Eickhoff et al. 2005) for anatomical identification of brain activation/deactivation.

#### Comparison between groups and conjunction analysis

To examine group-specific brain activation, we compared the images (imagery > baseline) obtained from the sighted group with those obtained from the blind group (sighted vs. blind). We also examined the opposite comparison (blind vs. sighted). To examine consistent brain activation/deactivation between groups (group-non-specific), we performed a conjunction analysis (Price and Friston 1997). In these analyses, we used the FWE-corrected extent threshold of *p* < 0.05 in the entire brain for a voxel-cluster image generated at the uncorrected height threshold of *p* < 0.001.

#### ROI analysis

Previous fMRI studies using a locomotor imagery task (Deutschländer et al. 2009a, b; Jahn et al. 2009) reported that the imagery of blind participants more strongly activated the multisensory vestibular areas (insular and superior temporal regions) and primary sensorimotor cortices (Deutschländer et al. 2009a, b), whereas that of sighted participants activated the parahippocampal and fusiform regions (Deutschländer et al. 2009a, b; Jahn et al. 2004; Jahn et al. 2008; Jahn et al. 2009). ROI analysis was performed to examine the replicability of these results. In this analysis, we extracted the beta value from the 14-mm radius sphere around a peak voxel from each region reported in the previous study (Deutschländer et al. 2009a) and plotted individual beta values for each group and each ROI separately (Fig. 3). For statistical evaluation, we performed a two-sample t-test for each ROI.

#### Functional connectivity analysis

Seed-based functional connectivity analysis was performed to identify brain regions in which activity co-varied with that in a seed region (Amemiya et al. 2019), which may not be detected by standard contrast analysis (Morita et al. 2014; Zaki et al. 2007) and may reveal more dynamic behavior of brain networks underlying motor imagery in sighted and blind participants.

In the conjunction analysis, six clusters of active voxels were identified in the bilateral supplementary motor areas (SMA) extending into right premotor cortex (PM) and right superior frontal gyrus (SFG), bilateral inferior frontal cortices (IFC), and left superior parietal lobule (SPL), which were consistently activated in both groups (Table 4). We identified 14 peaks in these clusters. Functional connectivity was analyzed for each of the 14 peaks (Table 4).

**Fig. 2:**
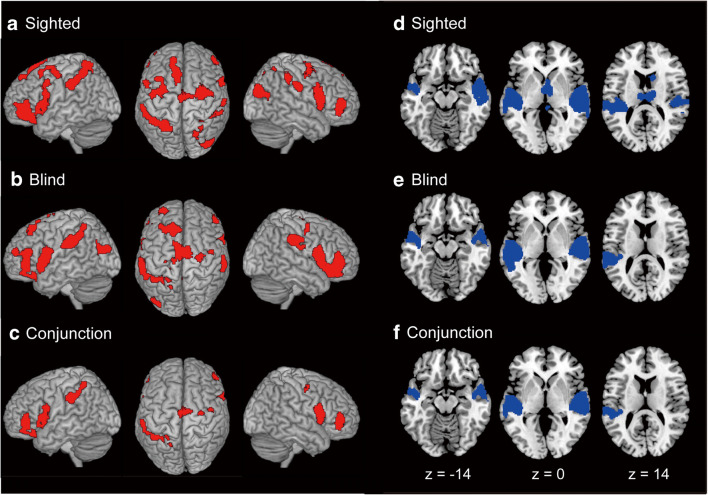
Brain regions active during locomotor imagery in sighted (**a**) and blind (**b**) groups, and common activation between groups revealed by conjunction analysis (**c**). We used the FWE-corrected extent threshold of *p* < 0.05 in the entire brain for a voxel-cluster image generated at the uncorrected height threshold of *p* < 0.001. In each row, brain activation (red sections) is rendered onto the MNI standard brain (left column, left view; middle column, top view; right column, right view). Brain regions deactivated during locomotor imagery in sighted (**d**) and blind (**e**) groups, and common deactivation between groups revealed by conjunction analysis (**f**). Deactivation (blue sections) is superimposed on the MNI standard anatomical image. Left panel in each row shows deactivation in the axial slice at MNI coordinates of z = −14, middle at z = 0, right at z = 14, respectively

We first extracted the time-course data for each participant obtained from the 14-mm radius sphere around each peak (seed region), which was identified in common activation (Fig. 2c). This radius was selected based on the final smoothness of the present functional imaging data. Then, to avoid the collinearity, the time course of a seed region was orthogonalized with respect to the task-related regressor with the following procedures. First, the time course was normalized by subtracting its (temporal) mean and divided by its standard deviation. Then, we applied Gram-Schmidt orthogonalization to the regressors by means of the function “spm_orth.m” implemented in the SPM toolbox. These orthogonalization was performed for each run in each participant. In the analysis, we constructed a second GLM (independent of the first GLM) for each seed region in each participant. The orthogonalized time-course data obtained from a seed region was included as a linear regressor in the design matrix for the GLM.

This GLM also contained an imagery-related regressor for the imagery epoch and the six realignment parameters as regressors of no interest. In general, during performance of a particular task, the BOLD signal includes both task-related and neuronal fluctuation components (Saito et al. 2010). Thus, the regressor (the time-course data obtained from a seed region) most likely contained both task-related and neuronal fluctuation components. In the present connectivity analysis, brain regions in which activity co-varied with activity changes (imagery-related and neuronal fluctuation components) in a seed region should have been identifiable, which could not be detected merely by the imagery-related regressor (simple boxcar function). Hence, the brain regions detected in this analysis were likely to share these activity components with the seed region.

The above analysis generated an individual image, which described brain regions where activity co-varied with the activity in each seed region. The images obtained from all participants were entered into the second-level random effects group analysis separately for each group. Second-level analyses were conducted by performing a *two-sample t-test between groups.* For statistical evaluation, we used the FWE-corrected extent threshold of *p* < 0.05 in the entire brain for a voxel-cluster image generated at the cluster-defining uncorrected height threshold of *p* < 0.001.

#### Post hoc analysis

Regardless of walking or jogging, imagery time was shorter in the blind group than in the sighted group, although the actual time did not differ between groups (Fig. 1a). This was an unexpected finding. To further investigate neuronal correlates associated with shorter imagery time in blind participants, correlation analysis was performed, excluding the data from two blind participants (see above). Using individual mean imagery time for walking and jogging as a covariate, we examined brain regions in which imagery-related activity was correlated with imagery time across the 12 blind participants. We used the FWE-corrected extent threshold of *p* < 0.05 in the entire brain for a voxel-cluster image generated at the uncorrected height threshold of p < 0.001.

Activity in the right inferior occipital region (38, −84, −4) positively correlated with imagery time. This region was closely located in the visual association area (36, −69, −14), which is activated during visually-dominant motor imagery in sighted people (Guillot et al. 2009; Fig. 5b). We extracted the beta value from the 14-mm radius sphere (ROI) around the peak (36, −69, −14) in each blind participant and plotted individual beta values against individual imagery times (Fig. 5c).

## Results

### Training

The participants were able to draw circle-like trajectories in both walking and jogging conditions, although many were imperfect circles. In the sighted group, the mean actual time across participants was 16.0 s (range: 12.6 to 19.9) and 11.6 s (range: 9.5 to 14.2) for walking and jogging, respectively (Fig. 1a). Likewise, the mean imagery time was 17.5 s (range: 12.0 to 23.4) for walking and 11.9 s (range: 6.3 to 16.5) for jogging. In the blind group, the mean actual time was 15.6 s (range: 11.1 to 20.0) and 11.0 s (range: 8.3 to 15.3), and the mean imagery time was 12.6 (range: 7.5 to 18.9) and 9.2 s (range: 6.8 to 12.1) for walking and jogging, respectively. Thus, both actual and imagery times for walking were substantially longer than those for jogging, and this was consistently observed in both groups (Fig. 1a). In contrast, imagery time was similar to actual time in the sighted group regardless of condition, whereas imagery time became shorter in the blind group in both conditions.

Three-way ANOVA revealed a significant interaction between three factors (Group (2) x Condition (2) x Task (2); *F*(1, 26) = 4.34; *p* = 0.047). There was a significant interaction between two factors (Group (2) x Task (2); *F*(1, 26) = 6.49; *p* = 0.017) and a main effect of Condition (walking, jogging; *F*(1, 26) = 194.78; *p* = 1.4 × 10^−13^).

Given the significant interaction between three factors, we performed two two-way ANOVAs. In the sighted group, a repeated measures two-way ANOVA for actual and imagery times revealed a significant main effect of Condition (walking, jogging; *F*(1, 15) = 104.05; *p* = 3.8 × 10^−8^) and no significant main effect of Task (actual, imagery; *p* = 0.33). This indicated that both actual and imagery times for walking were significantly longer than those for jogging (Fig. 1a). In contrast, in the blind group, ANOVA revealed significant main effects of Condition (walking, jogging; *F*(1, 11) = 125.39; *p* = 2.4 × 10^−7^) and Task (actual, imagery; *F*(1, 11) = 7.27; *p* = 0.021). This indicated that both actual and imagery times for walking were significantly longer than those for jogging, as observed in the sighted group. However, imagery time became significantly shorter than actual time regardless of condition in the blind group.

Two-way ANOVA revealed a significant interaction between Group and Task in the walking condition (*F*(1, 26) = 7.27; *p* = 0.012) and a trend for interaction in the jogging condition (*F*(1, 26) = 3.76; *p* = 0.063). Further between-group comparisons using a two-sample t-test revealed that in the walking condition, imagery time in the blind group was significantly shorter than that in the sighted group (*t*(26) = 3.61, *p* = 0.0026 corrected), although there was no significant difference in actual time between groups (*p* = 0.36). Likewise, in the jogging condition, imagery time in the blind group was significantly shorter than that in the sighted group (*t*(26) = 2.63, *p* = 0.028 corrected), with no difference in actual time between groups (*p* = 0.94).

Regardless of condition, imagery time was shorter in the blind group than in the sighted group, although actual time did not differ between groups (Fig. 1a).

Mean velocity of sighted participants was 0.89 m/s (range: 0.72 to 1.07) and 1.32 m/s (range: 1.03 to 1.65) for walking and jogging, respectively. Mean velocity of blind participants was 0.89 m/s (range: 0.59 to 1.07) and 1.47 m/s (range: 1.07 to 1.75) for walking and jogging, respectively. Jogging velocity was faster in the blind group than in the sighted group, while walking velocity was the same between groups (Fig. 1b).

Two-way ANOVA revealed a significant interaction between Group and Condition [*F*(1, 27) = 8.09; *p* = 0.0084]. Between-group comparison using two-sample t-test revealed that in the jogging condition, velocity in the blind group was significantly faster than that in the sighted group (*t*(27) = 2.54, *p* = 0.034 corrected). There was no significant difference in the walking condition between groups (*p* = 0.97).

### Subjective rating of motor imagery during fMRI scanning

Kinesthetic index value was significantly greater in the blind group than in the sighted group (*t*(28) = 2.28, *p* = 0.03; Fig. 1c), even when data from the congenital and early blind participants were excluded. We confirmed that the kinesthetic index value was not associated with either the onset or duration of total blindness (*N* = 14; *r* = −0.04, *p* = 0.89 for onset; *r* = 0.21, *p* = 0.47 for duration).

### Brain activation

The present locomotor (walking and jogging) imagery activated a broad range of cortical and subcortical brain structures in both groups (Fig. 2a, b), with no significant activation in the primary motor cortex (M1). Activation peaks in the sighted and blind groups are tabulated in Tables 2 and 3, respectively. The patterns of brain activation were not significantly different between groups.

**Table 2:** Brain activations in sighted group

Clusters	Size	MNI coordinates			*T* -value	Anatomical identification (cytoarchitectonic area)
		x	y	z		
Left hemisphere						
IFG cluster	1841	−48	14	4	6.60	Area 44
		−38	42	−18	5.48	pars Orbitalis
		−46	42	−2	5.03	pars Triangularis
		−56	10	36	4.44	Precentral gyrus
		−42	54	6	3.76	MFG
SFG cluster	722	−8	26	66	4.90	Posterior medial frontal
		−14	44	46	4.43	SFG
		−10	30	36	4.21	Superior medial gyrus
Parietal cluster	1303	−28	−54	60	5.35	Area 7PC
		−58	−30	36	5.01	Area PFt
		−42	−50	52	4.96	Area hIP2
Right hemisphere						
Anterior IFG cluster	370	52	44	−2	5.31	pars Triangularis
Posterior IFG cluster	1061	50	12	8	6.16	Area 44
		40	4	2	4.38	Insula lobe
Bilateral PM/SMA cluster	2472	40	−6	48	5.64	Right PM
		24	−6	60	5.31	SFG
		−34	−6	50	5.16	Left PM
		4	−6	60	4.19	Right SMA
		−12	−6	56	4.09	Left SMA
		−40	−12	54	3.96	MFG
IPL cluster	643	44	−38	44	5.38	Area hIP2
		58	−22	32	5.04	Area PFt
		60	−34	48	4.09	Area PF
SPL cluster	481	24	−56	52	5.52	Area hIP3
Occipital cluster	644	44	−78	24	5.26	Area PGp
		30	−76	30	4.34	MOG
		44	−62	12	3.83	MTG

Height threshold, p < 0.001 uncorrected; extent threshold, p < 0.05, FWE-corrected across the entire brain. Size = number of active voxels. For anatomical identification of peaks, we only considered cytoarchitectonic areas available in the anatomy toolbox that had a greater than 30% probability. The cytoarchitectonic area that had the highest probability was reported for each peak. When no cytoarchitectonic area with more than 30% probability was available to use for determination of the peak, we instead provided the anatomical location of the peak. In each cluster, we reported peaks that were more than 14 mm apart from each other listed in descending order of T-values. Abbreviations: IFG, inferior frontal gyrus; MFG, middle frontal gyrus; SFG, superior frontal gyrus; PM, premotor cortex; SMA, supplementary motor area; IPL, inferior parietal lobule; SPL, superior parietal lobule; MOG, middle occipital gyrus; MTG, middle temporal gyrus

**Table 3:** Brain activations in blind group

Clusters	ASize	MNI coordinates			*T* -value	Anatomical identification (cytoarchitectonic area)
		x	y	z		
Left hemisphere						
Anterior IFG cluster	1389	−32	24	−22	5.62	pars Orbitalis
		−48	38	0	5.46	pars Triangularis
		−38	40	16	5.42	MFG
Posterior IFG cluster	2124	−38	2	4	9.11	Area 44
		−26	−14	2	5.49	Putamen
IPL cluster	963	−60	−28	32	6.17	Area PFop
		−56	−44	46	4.58	Area PF
SPL cluster	569	−24	−46	50	5.67	Area 5 L
		−42	−48	56	4.42	Area 7PC
Occipital cluster	377	−36	−68	22	5.15	MOG
		−38	−86	24	5.11	Area PGp
Right hemisphere						
IFG cluster	3509	50	40	−6	6.75	6.75 pars Orbitalis
		62	12	12	5.93	Area 44
		28	−12	6	5.44	Putamen
		42	38	10	5.14	pars Triangularis
		38	40	−16	4.84	MOG
Bilateral PM/SMA cluster	1936	24	−15	58	5.83	Right PM
		−12	4	62	5.40	Left SMA
		8	−8	66	4.89	Right SMA
		−18	−16	58	3.88	Left PM
IPL cluster	432	62	−26	32	4.63	Area PFt
		54	−14	26	3.77	Area 3b
Cerebellar cluster	298	16	−40	−26	4.87	Lobule V

Height threshold, p < 0.001 uncorrected; extent threshold, p < 0.05, FWE-corrected across the entire brain. Size = number of active voxels. For anatomical identification of peaks, we only considered cytoarchitectonic areas available in the anatomy toolbox that had a greater than 30% probability. The cytoarchitectonic area that had the highest probability was reported for each peak. When no cytoarchitectonic area with more than 30% probability was available to use for determination of the peak, we instead provided the anatomical location of the peak. In each cluster, we reported peaks that were more than 14 mm apart from each other listed in descending order of T-values. Abbreviations: IFG, inferior frontal gyrus; MFG, middle frontal gyrus; MOG, middle occipital gyrus; PM, premotor cortex; SMA, supplementary motor area; IPL, inferior parietal lobule; SPL, superior parietal lobule

We observed common brain activation patterns between groups. Conjunction analysis revealed common brain activation in the bilateral SMA extending into the right PM, bilateral inferior frontal cortices, and left IPL and SPL (Fig. 2c). Peaks in common activation are summarized in Table 4.

**Table 4:** Common brain activations

Clusters	Size	MNI coordinates			*T* -value	Anatomical identification (cytoarchitectonic area)
		x	y	z		
Left hemisphere						
Anterior IFG cluster	559	−38	42	−18	5.41	pars Orbitalis
		−48	40	−2	5.03	pars Triangularis
Posterior IFG cluster	705	−48	14	4	6.60	Area 44
		−38	16	−8	3.70	Insula lobe
SPL cluster	341	−26	−50	54	4.93	Area 2
		−42	−48	56	4.42	Area 7PC
Right hemisphere						
Anterior IFG cluster	331	52	42	−6	5.22	pars Orbitalis
		48	44	8	4.61	MFG
Posterior IFG cluster	756	50	12	8	6.16	Area 44
		40	4	2	4.38	Insula lobe
PM-bilateral SMA cluster	674	42	−8	46	5.23	PM
		26	−14	60	5.00	SFG
		4	−12	58	4.13	Right SMA
		−10	−4	58	3.70	Left SMA

Height threshold, p < 0.001 uncorrected; extent threshold, p < 0.05, FWE-corrected across the entire brain. Size = number of active voxels. For anatomical identification of peaks, we only considered cytoarchitectonic areas available in the anatomy toolbox that had a greater than 30% probability. The cytoarchitectonic area that had the highest probability was reported for each peak. When no cytoarchitectonic area with more than 30% probability was available to use for determination of the peak, we instead provided the anatomical location of the peak. In each cluster, we reported peaks that were more than 14 mm apart from each other listed in descending order of T-values. Abbreviations: IFG, inferior frontal gyrus; MFG, middle frontal gyrus; SFG, superior frontal gyrus; PM, premotor cortex; SMA, supplementary motor area; SPL, superior parietal lobule. We used each of 14 peak voxels as seed voxels in the connectivity analysis

### Brain deactivation

We observed significant deactivation during locomotor imagery. Deactivation peaks are summarized in Table 5. Imagery in the sighted group significantly deactivated the bilateral superior temporal gyri (STG), part of the posterior-insula, temporal poles (TP), and thalamus; and right Heschls gyrus, caudate, and cerebellar vermis (Fig. 2d). Imagery in the blind group deactivated the bilateral STG, part of the posterior-insula, and TP; and left MTG (Fig. 2e). Conjunction analysis disclosed common deactivation in the bilateral STG, part of the posterior-insula, TP; and left MTG between groups (Fig. 2f).

**Table 5:** Brain deactivations

Clusters	Size	MNI coordinates			*T* -value	Anatomical identification (cytoarchitectonic area)
		x	y	z		
Brain deactivations in sighted group						
Left temporal cluster	3109	−56	−12	−2	10.55	STG
		−46	2	−16	4.35	TP
Right temporal cluster	3824	48	−16	4	10.72	Area TE 1.0 (Heschl’s gyrus)
		40	8	8–24	3.61	TP
Subcortical cluster	1719	6	−10	4	5.98	Right thalamus (prefrontal)
		12	14	16	5.38	Right caudate nucleus
		−6	−6 -14	14	4.37	Left thalmus (temporal)
		2	−36	−6	4.24	Right cerebellar vermis
Brain deactivations in blind group						
Left temporal cluster	3525	−60	−22	0	10.63	MTG
		−60	−2	−10	7.66	Area TE 3
		−42	0	−20	9.48	TP
Right temporal cluster	2569	48	−18	4	9.48	Area TE 1.0
		66	−12	−6	7.04	Area TE 3
		48	10	−20	4.77	TP
Common brain deactivations						
Left temporal cluster	2620	−58	−20	0	10.08	MTG
		−46	−28	6	9.56	STG
		−60	−4	−8	7.45	Area TE 3
		−46	2	−16	4.35	TP
Right temporal cluster	2440	48	−18	4	9.48	Area TE 1.0
		66	−14	−4	6.83	Area TE 3
		50	10	−18	4.59	TO

Height threshold, p < 0.001 uncorrected; extent threshold, p < 0.05, FWE-corrected across the entire brain. Size = number of active voxels. For anatomical identification of peaks, we only considered cytoarchitectonic areas available in the anatomy toolbox that had a greater than 30% probability. The cytoarchitectonic area that had the highest probability was reported for each peak. When no cytoarchitectonic area with more than 30% probability was available to use for determination of the peak, we instead provided the anatomical location of the peak. In each cluster, we reported peaks that were more than 14 mm apart from each other listed in descending order of T-values. Abbreviations: STG, superior temporal gyrus; TP, temporal pole; MTG, middle temporal gyrus

### ROI analysis

**Fig. 3:**
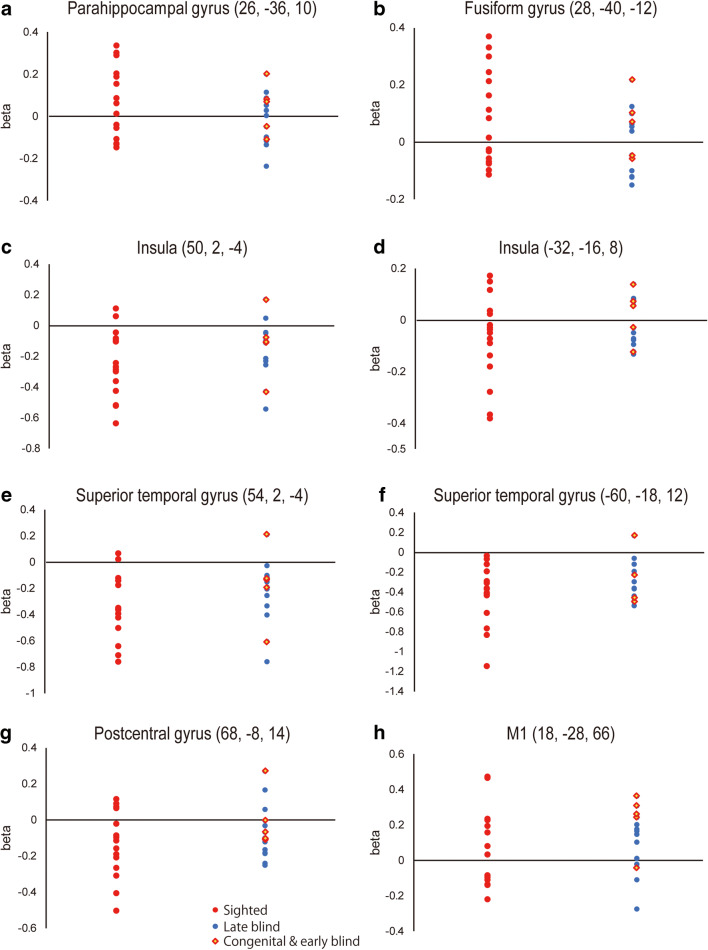
Results of ROI analysis. Individual beta values were plotted in each group and in each ROI separately. ROIs were selected based on a previous report (Deutschländer et al. 2009a). **a** right parahippocampal gyrus. **b** right fusiform gyrus. In the previous study, the sighted group showed significantly greater activity during locomotor imagery than did the blind group in these regions, which was not the case in the present study. **c** right insula. **d** left insula. e: right superior temporal gyrus. **f** left superior temporal gyrus. **g** precentral gyrus. **h** right M1. In the previous study, the blind group showed significantly greater activity than that of the sighted group in these regions, which was not the case in the present study. Red filled dots represent beta values for sighted participants, blue for late blind participants, and red diamond for congenital and early blind participants

No significant group differences for any ROI in the present study were observed (Fig. 3), in contrast to previous reports (Deutschländer et al. 2009a, b; Jahn et al. 2009).

### Group differences in functional connectivity

Despite no significant group differences in the degree of brain activation, significant group differences in functional connectivity analyses were observed. The results are summarized in Table 6.

**Table 6:** Group difference in functional connectivity with seed regions

Seed (x, y, z)	Size	MNI coordinates			*T* -value	Anatomical identification (cytoarchitectonic area)
		x	y	z		
Sighted > Blind						
Right SMA (4, −12, 58)	1412	−12	−102	−2	4.76	Left area 17
		12	−98	−4	3.85	Right area 17
Blind > Sighted						
Left SMA (4, −12, 58)	440	10	−50	−50	5.60	Right lobule IX (Hem)
		28	−60	−50	4.55	Right cerebellum
Right SFG (26, −14,60)	409	18	−56	−44	5.48	Right cerebellum
		26	−46	−50	4.28	Right lobule VIIIb (Hem)
		8	−44	−50	4.01	Right cerebellum

Height threshold, p < 0.001 uncorrected; extent threshold, p < 0.05, FWE-corrected across the entire brain. Size = number of active voxels. For anatomical identification of peaks, we only considered cytoarchitectonic areas available in the anatomy toolbox that had a greater than 30% probability. The cytoarchitectonic area that had the highest probability was reported for each peak. When no cytoarchitectonic area with more than 30% probability was available to use for determination of the peak, we instead provided the anatomical location of the peak. In each cluster, we reported peaks that were more than 14 mm apart from each other listed in descending order of T-values. Abbreviations: SMA, supplementary motor area; SFG, superior frontal gyrus

**Fig. 4:**
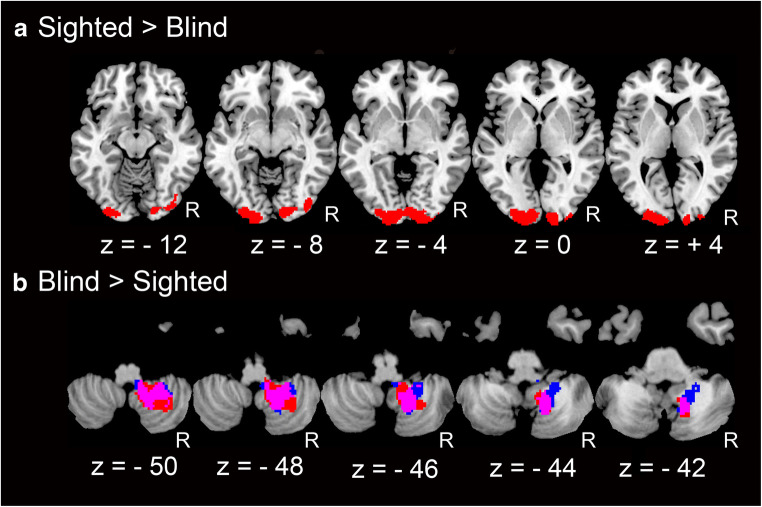
Functional connectivity results. We used the FWE-corrected extent threshold of *p* < 0.05 in the entire brain for a voxel-cluster image generated at the cluster-defining uncorrected height threshold of *p* < 0.001. **a** Brain regions (red) that showed significantly greater functional coupling with right SMA seed region (14-mm radius sphere around [4, −12, 58]) in the sighted group compared to that in the blind group (sighted > blind). The regions are superimposed on five axial slices (z = −12, −8, −4, 0 and + 4) of the MNI standard anatomical image. **b** Brain regions that showed significantly greater functional connectivity with left SMA (14-mm radius sphere around [−10, −4, 58]; red) and with right SFG (14-mm radius sphere around [26, −14, 60]; blue) in the blind group compared to that in the sighted group (blind > sighted). Purple regions show overlap between them. Clusters in five axial slices (z = −50, −48, −46 − 44 and − 42) is shown

The group difference was observed in brain regions whose activity co-varied with that of the SMA cluster. The bilateral primary visual cortices (area 17) showed significantly stronger functional coupling with the right SMA (seed voxel at (4, −12, 58); Fig. 4a) in the sighted group when compared with that in the blind group. In contrast, in the blind group, the right cerebellum (Lobules IX and VIIIb) exhibited significantly stronger functional connectivity with the left SMA (seed voxel at (−10, −4, 58); Fig. 4b, red) and the right SFG (seed voxel at (26, −14, 60); Fig. 4b, blue) compared to that in the sighted group. No significant group differences were observed in connectivity with the remaining seed voxels listed in Table 4.

### Post hoc analysis

**Fig. 5:**
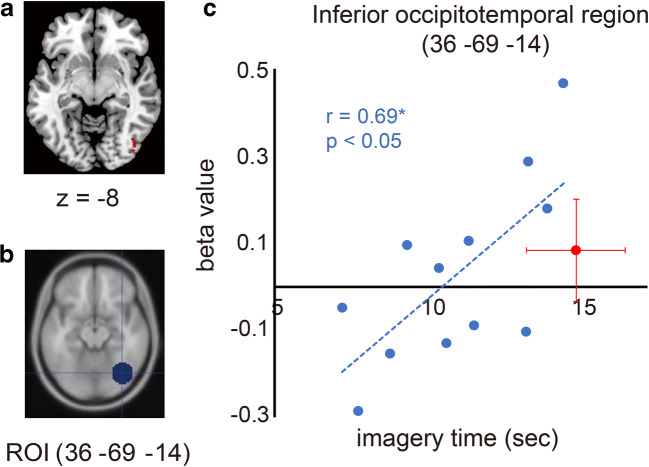
**a** Brain region (red) in which activity correlated positively with imagery time in the blind group. The activity is superimposed on an axial slice (z = −8) of the MNI standard anatomical image. **b** Region of interest (ROI; blue) is displayed on an axial slice (z = −14) of the MNI standard mean anatomical image. We defined ROI as a 14-mm radius sphere around the peak (36, −69, −14), which was reported to be activated during visually dominant motor imagery in a previous study (Guillot et al. 2009). **c** Relationship between individual beta values (vertical axis) and individual imagery times (horizontal axis) in the blind group. Each blue dot represents the data obtained from each blind participant. Dashed lines indicate a linear regression line fitted to the data. ROI activity correlated positively with imagery time (*N* = 12, *r* = 0.69, *p* = 0.013). As a reference, we also plotted the mean value (red dot) across all sighted participants. Red bars indicate the standard deviation of the means across these participants

We investigated the neuronal correlates associated with shorter imagery time in blind participants (Fig. 1a). Activity in the right inferior occipital region (voxel size = 286, peak in right area hOc4lp [38, −84, −4], T = 5.14) correlated positively with imagery time (Fig. 5a). This activity was closely located in the visual association area (36, −69, −14), which was previously reported to be activated during visually dominant motor imagery in sighted people (Guillot et al. 2009; Fig. 5b). When we plotted individual brain activity around this region against individual imagery times (Fig. 5c), we observed that activity in this region correlated positively with imagery time (*N* = 12, *r* = 0.69, *p* = 0.013; Fig. 5c).

## Discussion

In the present study, we examined neurological and behavioral features of locomotor imagery in blind participants. We found novel evidence that imagery time becomes shorter in blind participants during training. fMRI results indicated that spatio-visual components more easily intruded into imagery in the sighted group, while blind participants were capable of having relatively pure kinesthetic motor imagery as per instructions. In addition, distinct patterns of functional connectivity with the SMA were observed between sighted and blind participants, although no significant group differences in degree of brain activation was detected. In the sighted group, the visual cortices showed stronger functional connectivity with the SMA; whereas in the blind group, the right cerebellum was strongly functionally coupled with the SMA.

### Limitations of the study

A limitation of the present study was the small number of blind participants (three congenital, two early, and nine late blind participants). This precluded differences between congenital/early and late blind participants, although previous studies have suggested that differences exist (Collignon et al. 2013; Deutschländer et al. 2009a; Sadato et al. 2002).

### Different characteristics of motor imagery in blind participants

In both groups, the imagery time for walking was significantly longer than that for jogging, as the actual time for walking was significantly longer than that for jogging (Fig. 1a). The longer actual time for walking than jogging was compatible with the fact that walking velocity was significantly slower than jogging velocity in both groups (Fig. 1b).

In the sighted group, imagery time was similar to actual time regardless of condition (Fig. 1a), suggesting that locomotor imagery obeyed the same motor rules as those in an actual locomotor task, fitting the general notion that imagined movements obey the same motor rules and biomechanical constraints as those of real movements, as suggested by many psychological studies (Decety and Jeannerod 1995; Jeannerod 1994; Parsons 1987; Sirigu et al. 1996; Papaxanthis et al. 2002; Decety et al. 1989; Parsons 1994).

In contrast, in the blind group, imagery time was significantly shorter than actual time in both conditions, and imagery time was significantly shorter than that in the sighted group, although actual time did not differ between groups (Fig. 1a). This behavioral evidence suggested that motor imagery in the blind and sighted groups had distinct characteristics, indicating distinct neuronal substrates as suggested previously (Deutschländer et al. 2009a; Imbiriba et al. 2006; Imbiriba et al. 2013).

How can these behavioral observations be explained? One possibility specific for the present circular locomotion task is that the size of mentally represented circles in blind participants could be limited and smaller, such that the time required to imagine completing locomotion around a full circle was shorter. This view is compatible with the notion that the sense of spatial extent and depth is generally limited or lacking when congenitally blind people imagine space, probably due to lack of information about one’s surrounding environment which is normally captured by the visual system (Arditi et al. 1988). This also agrees with previous findings that circular trajectories in early blind people tend to be compressed when they reproduce circular walking experienced through auditory-navigation (Gori et al. 2017).

A trend of shorter motor imagery (first-person perspective imagery of arm, leg, and whole-body movements) compared to that of actual execution has been reported in congenitally blind people (10% shorter in the blind, and only 1% shorter in sighted people; (Imbiriba et al. 2013). Hence, other explanations may underpin shorter motor imagery in blind people.

The limited ability to visualize events and environments associated with imaginary actions may be associated with this behavioral phenomenon in the blind. Indeed, we observed that activity in the right inferior occipitotemporal region positively correlated with imagery time across participants in the blind group (Fig. 5). This result suggested that blind participants who reported shorter imagery time did not substantially recruit higher-order visual areas during motor imagery. The inferior occipitotemporal region can be activated when transcranial magnetic stimulation is provided to the primary somatosensory cortex of blind people (Wittenberg et al. 2004) and M1 of sighted people (Hanakawa et al. 2009), indicating that higher-order visual areas are connected with the sensory-motor system. We conjecture that recruiting higher-order visual areas may influence subjective experience during motor imagery, which may then affect quantitative measures (e.g., duration) of motor imagery in blind people, but more evidence is required to support this claim.

### Similarities and differences to previous studies

Our results indicated that locomotor imagery in blind people was qualitatively different from that of sighted people, which is compatible with previously reported conclusions (Deutschländer et al. 2009a). However, the present results partly disagree with previous findings, even though both studies used locomotor imagery tasks.

In a previous study, congenitally blind and sighted participants imagined walking and running, which were experienced before the fMRI experiment. Locomotor imagery in sighted participants more strongly activated the parahippocampal and fusiform regions compared with that in blind participants, whereas imagery in blind participants more strongly activated the multisensory vestibular areas (posterior insula and adjacent superior temporal cortices) and primary sensorimotor cortices (SM1) compared to that in sighted participants (Deutschländer et al. 2009a). In the present study, we did not detect significant group differences even after assessing brain activity in each region reported previously (Fig. 3). One possible explanation for this is the difference in sensory experiences imagined by participants, which likely depended on those during the prescanning training (see details in Introduction and Methods). Parahippocampal and fusiform activation during locomotor imagery in the sighted group was likely associated with imagination of locomotor spatial navigation and the visual environment, which were experienced during training with their eyes open (Deutschländer et al. 2009a). Such activation has been consistently reported by the same group, which consistently use the same pre-fMRI training (la Fougère et al. 2010; Jahn et al. 2004; Wagner et al. 2008; Zwergal et al. 2012). Parahippocampal activity is also reported when sighted volunteers imagine virtual walking through a corridor (Iseki et al. 2008). The lack of significant parahippocampal and fusiform activation in the present sighted group may be associated with lack of visual experiences during training.

In the present sighted group, we observed significant deactivation mainly in bilateral superior temporal gyri (areas TE 1.0, 2, 3) during motor imagery (Figs. 2d and 3e, f). Adjacent bilateral posterior-insular cortices were also partly deactivated (Fig. 3c). Thus, the present deactivation was observed mainly in auditory regions, auditory association areas (Morosan et al. 2001), and partly in adjacent vestibular cortices (Bense et al. 2001; Eickhoff et al. 2006; Frank et al. 2014). Similar patterns of deactivation have been consistently reported during locomotor imagery in sighted participants (la Fougère et al. 2010; Wagner et al. 2008; Jahn et al. 2004; Zwergal et al. 2012). It is assumed that the suppression of brain activity in putative auditory and vestibular regions may avoid cross-modal distraction and interference from these sensory modalities to kinesthetically focused locomotor imagery, as proposed by previous studies (Deutschländer et al. 2009a; la Fougère et al. 2010; Jahn et al. 2004; Zwergal et al. 2012).

Despite these similarities, a discrepancy was that these regions (especially posterior-insular vestibular regions) were activated in the blind group in a previous study (Deutschländer et al. 2009a) but were deactivated in the present blind group, as observed in the sighted group (Figs. 2e, 3c, and Table 5). In animals, the vestibular system becomes active during locomotion to ensure maintenance of equilibrium (Marlinsky 1992). Thus, the present circular locomotion task should have activated this system particularly in the blind group who rely more on vestibular information than do sighted people. However, we did not observe posterior-insular vestibular activation even in this group. The reasons for this discrepancy are unknown but may be underpinned by differences in participants and imagery tasks between studies.

Finally, we did not observe significant SM1 activation during imagery in the blind group compared to that in the sighted group (Fig. 2a, b), in contrast to previous findings (Deutschländer et al. 2009a), In general, SM1 activation during motor imagery could be affected by many factors including individual differences or degree of neuronal suppression of motor commands that may affect possible muscle activity (Hétu et al. 2013; Kasess et al. 2008). Moreover, it is generally believed that fMRI is not sensitive enough to detect subtle activity changes in SM1 during motor imagery. Indeed, according to a meta-analysis (Hétu et al. 2013), the majority (82%) of previous motor imagery studies have reported no SM1 activation even when participants are instructed to generate kinesthetic motor imagery, which aligns with our findings.

### Functional connectivity

The brain regions commonly activated in both sighted and blind groups (Fig. 2c and Table 4) are consistently reported to be active during various types of motor imagery tasks including locomotor imagery (Hétu et al. 2013). Hence, these common brain regions can be regarded as core brain structures that are active during motor imagery in general. These consistent findings indicate the generalizability of the present results, although the number of participants in each group may have been insufficient (Thirion et al. 2007). In addition, a meta-analysis reported similar patterns of brain activation during gait motor imagery of sighted people (Hétu et al. 2013), further indicating the importance of these brain structures in locomotor imagery.

In a previous study (Guillot et al. 2009), kinesthetic motor imagery was reported to more strongly activate the SMA, inferior frontal cortices (area 44), IPL, and cerebellum, while visual motor imagery more strongly activated the PM, SPL, and visual cortices relative to each other, although the SMA and PM were activated during both kinesthetic and visual motor imagery (Hétu et al. 2013). Since the commonly activated brain regions identified here included many of these brain structures (Fig. 2c and Table 4), it is likely that motor imagery in sighted and blind groups contained both kinesthetic and spatio-visual components, which is compatible with subjective reports (Fig. 1c).

Among the common brain regions (Fig. 2c), the SMA was the only region that consistently showed group-specific functional connectivity with other regions in both groups (Fig. 4). Thus, the SMA appears to be a particularly important brain node during locomotor imagery in both sighted and blind participants. Indeed, in sighted participants, the SMA has been reported to be active not only during locomotor imagery (Jahn et al. 2008; Jahn et al. 2004; Miyai et al. 2001; la Fougère et al. 2010; Wagner et al. 2008; Wang et al. 2009; Malouin et al. 2003; Zwergal et al. 2012) but also during real locomotion (Miyai et al. 2001; Fukuyama et al. 1997; Hanakawa et al. 1999).

In the present connectivity analysis, we found that bilateral primary visual cortices were strongly coupled with the right SMA in the sighted group, while the right cerebellum was strongly coupled with the left SMA in the blind group (Fig. 4 and Table 6). Our regressor (the time-course data obtained from a seed region) most likely contained not only task-related fluctuations, but also non-task related fluctuations, which can be included in resting-state brain activity. Unfortunately, we could not measure resting-state brain activity in the present study. However, a previous study reported lower resting-state functional connectivity between motor (e.g., SMA) and visual areas in early blind people (Yu et al. 2008). Therefore, the lower functional connectivity between motor (SMA) and visual areas in the blind participants (higher in sighted group) could be affected by their potentially lower motor-visual resting-state connectivity. In addition, there is a possibility that this lower motor-visual connectivity in blind people could be due to top-down suppression of visual cortices, which is likely to be experienced by blind (especially non-congenital) people (Castaldi et al. 2019).

The presently reported stronger SMA-cerebellar connectivity in the blind has never been reported previously. This may be related to blind-specific motor imagery processes. Since the SMA and cerebellum are kinesthetically dominant motor imagery regions (Guillot et al. 2009), functional coupling between the SMA and cerebellum in the blind group (Fig. 4b) could be associated with their more kinesthetic motor imagery (Fig. 1c), although we found no significant correlation between the degree of functional coupling and kinesthetic index.

In this study, the right cerebellar region was slightly different from the regions reported in previous locomotor imagery studies (Jahn et al. 2008; la Fougère et al. 2010; Jahn et al. 2004; Wagner et al. 2008; Zwergal et al. 2012). The cerebellar lobule VIII can be considered one of the cerebellar sections involving higher-order sensorimotor processing (Guell et al. 2018; Stoodley and Schmahmann 2009). In addition, lobules VIII and IX are involved in voluntary leg placement and balance control of gait (Ilg et al. 2008). Thus, these cerebellar lobules seem to participate in higher-order sensorimotor processing associated with gait control. Hence, the coupling between SMA and these cerebellar regions during motor imagery indicates that blind participants may emulate higher-order sensorimotor processes associated with gait control during locomotor imagery.

In the neuroimaging literature, it is known that visual cortices in blind people may activate during neuronal processing of other sensory modalities (Kupers and Ptito 2014; Théoret et al. 2004). The present results suggest that this is not the case in motor imagery in blind people. The present lower functional coupling between SMA and visual cortices in blind participants could be related to weaker long-distance resting-state functional connectivity of visual cortices (Qin et al. 2015) and gray matter volume atrophy of visual cortices (Pan et al. 2007; Leporé et al. 2010; Modi et al. 2012) reported in blind people. Indeed, in the present study, we confirmed gray matter volume atrophy in visual cortices in blind participants compared with that in sighted participants (Supplementary Figure 1 and Supplementary Table 1). The visual regions with lower functional coupling in blind participants (Fig. 4a) substantially overlapped with the visual cortices where gray matter volume was significantly reduced (Supplementary Figure 1), although no significant correlation was observed between the degree of functional coupling and degree of atrophy across participants. Thus, an anatomical basis underscored by long-term brain plasticity may underlie the lower functional connectivity of visual cortices in blind people.

## Conclusions

We demonstrated neurological and behavioral features of locomotor imagery in blind people. This study provided valuable knowledge on the neural underpinnings of motor imagery in blind people, which may promote better understanding of their mental processes.

## Electronic supplementary material

ESM 1 (DOCX 115 kb)
