# Supplementary material for: Neurological and behavioral features of locomotor imagery in the blind
Source: Brain Imaging Behav. 2020 Apr 2;15(2):656–76. doi: 10.1007/s11682-020-00275-w (PMC8032591; doi:10.1007/s11682-020-00275-w)
Supplement: Supplementary file 1 — (DOCX 115 kb) [file 11682_2020_275_MOESM1_ESM.docx]

**Supplementary Material**

***Controllability of motor imagery (CMI) test***

First, using a controllability of motor imagery (CMI) test, we evaluated each participant’s ability to generate, manipulate, and hold imagined postures of their body parts in response to verbal instructions about sequential movements of body parts (left or right arm, left or right leg, torso, head; Naito, 1994; Nishida et al., 1986). The original CMI test is composed of 15 sets of trials (Nishida et al., 1986). However, for the present study, we selected nine sets of trials because our pilot study revealed that the expected final postures (see below) in the remaining six trials were very difficult for blindfolded participants to physically demonstrate.

One trial consisted of six consecutive verbal instructions, each of which instructed a movement of each body part (e.g., Step 50 cm forward on your right leg, bring your right arm 90 degrees to the front, and so on; see details in Naito, 1994). During the CMI test, blindfolded participants (see text) were required to imagine, from a first-person (egocentric) perspective, that they were actually moving their body parts according to the instructions, while they were seated. During the process of imagining, no actual movement was permitted. Each instruction was given every 3 sec. Starting from a basic posture (e.g., Stand straight with both your feet together), the participants had to keep constructing (updating) their entire imagined body posture by adding a new posture of one body part as per the instructions. Immediately after completion of the six instructions, participants had to physically demonstrate the final posture they had in their minds by actually performing it. We video-recorded their final postures.

In an off-line analysis, we gave a score when the final posture for each of the six body parts (left or right arm, left or right leg, torso, head) was correct. Hence, the highest possible score in the present CMI test was 54. The validity and reliability of this test were carefully evaluated in a previous study (Nishida et al., 1986). Further, it has been shown that the CMI score is higher in physically active athletes than in non-athletes (Naito, 1994). In addition, people who are good at having kinesthetic type of motor imagery score higher in the CMI test (Naito et al., 2002). Hence, the CMI test can be used as a unique and reliable measure to evaluate individual ability to generate, manipulate, and construct motor imagery from the first-person perspective, in general.

The average CMI score across participants was 44.8 (ranging from 35 to 53) and 41.7 (ranging from 24 to 54) for the sighted and blind groups, respectively. No difference was observed between groups (*df* = 28, *t* = 0.8, two-sample t-test *p* = 0.26), indicating that the controllability of motor imagery evaluated by this test is similar between the two groups.

In the present study, in our recruitment of blind participants, we contacted the Japanese Blind Football Association. The blind participants from our study included six blind soccer players (mean age, 31.7 ± 5.8 years; soccer experience more than 2 years) and eight age-matched (mean age, 33.3 ± 7.7 years) blind persons with no intensive experience of playing soccer in their lives. In order to match this participant composition for the sighted group, we also recruited eight soccer players (mean age, 32.4 ± 7.1 years; soccer experience more than 9 years) and eight persons with no intensive experience of playing soccer. As reported in our previous study (Naito, 1994), the CMI score was significantly higher in the soccer group (both sighted and blind participants; n = 14, mean CMI score = 46.9, ranging from 35 to 54) than in the non-soccer group (n = 16, mean CMI score = 40.1, ranging from 24 to 53; *df* = 28, *t* = 2.91, two-sample t-test *p* = 0.007).

***Voxel-based morphometry (VBM) analysis***

In the present study, we also performed a voxel-based morphometry (VBM) analysis to explore possible expansion and reduction in gray matter (GM) volume in the blind participants (n = 14) as compared to the sighted participants (n = 16). This may promote understanding as to how the long-term sensory information processing style of the present blind participants shapes anatomical features of their brains (c.f. Zatorre et al., 2012).

**Methods**

*MRI acquisition*

For each participant, a T1-weighted magnetization-prepared rapid gradient echo (MP-RAGE) image was also acquired in the same MR scanner. The imaging parameters were as follows: TR =1900 msec; TE = 2.48 msec; FA = 9º; FOV = 256 × 256 mm; matrix size =256 × 256 pixels; slice thickness = 1.0 mm; Voxel size = 1 × 1 × 1 mm; and 208 contiguous transverse slices.

*Image analysis*

First, we conducted visual inspection of the anatomical images obtained from all participants and confirmed the absence of observable structural abnormalities and motion artefacts in the images. These data were processed using Statistical Parametric Mapping (SPM12, Wellcome Trust Centre for Neuroimaging) and the VBM8 toolbox (http://dbm.neuro.uni-jena.de) implemented in the SPM12. All steps were carried out as recommended by Ashburner (2010) (http://www.fil.ion.ucl.ac.uk/~john/misc/ VBMclass10.pdf) (Ashburner, 2010). First, the anatomical image obtained from each participant was segmented into gray matter (GM), white matter (WM), cerebrospinal fluid (CSF), and non-brain parts (using new segmentation). In this process, we used the East Asian Brain template for anatomical normalization (affine regularization). Default settings in SPM12 were used for the remaining parameters.

Next, using the diffeomorphic anatomical registration through exponentiated Lie algebra (DARTEL) (Ashburner, 2007), we generated GM and WM DARTEL templates based on the anatomical images obtained from all participants (both sighted and blind). We used the DARTEL technique implemented in SPM12 as default settings. We then applied an affine transformation to the GM and WM DARTEL templates to align them with their tissue probability maps in MNI standard space. A segmented GM image from each participant was then non-linearly warped to the GM DARTEL template in MNI space (spatial normalization). The warped image was modulated by Jacobian determinants of the deformation field to preserve relative GM volume even after spatial normalization. The modulated image in each participant was smoothed with an 8-mm FWHM Gaussian kernel, and resampled to a resolution of 1.5 × 1.5 × 1.5 mm voxel size.

The preprocessed GM images obtained from all participants were then analyzed using voxel-wise statistical parametric mapping. Using two-sample t-tests, we identified brain regions that showed between-group differences in GM volume (sighted vs. blind and blind vs. sighted, respectively). In this analysis, we used age, handedness, presence or absence of physical training, and total brain volume (*i.e.,* sum of GM and WM volumes) as nuisance covariates (i.e., effects of no-interest). To exclusively select true positive GM voxels by eliminating possible noise lying outside the brain and to restrict the search volume, we generated a mask based on our present data using the Masking Toolbox (Ridgway et al., 2009) (<http://www0.cs.ucl.ac.uk/staff/g.ridgway/masking/>). Thus, voxels outside of this mask were excluded from the analysis. We reported brain regions that showed expansion and reduction of GM volume in the entire brain of the blind participants compared with those of sighted participants (> 143 voxels [expected voxels per cluster; Friston et al., 1994] in a voxel-cluster image using a height threshold of *p* < 0.005 uncorrected).

**Results and Discussion**

We found expansion of GM volume (blind > sighted) in the left superior temporal gyrus (cytoarchitectonic area TE3), right precentral rolandic opercular region including area 44, right amygdala and hippocampal region, and broader bilateral medial frontal regions (Supplementary Table 1 and Supplementary Figure 1).

In contrast, we found that the GM volume of the bilateral occipital cortices was remarkably reduced in the blind group compared to that in the sighted group (GM volume atrophy; Supplementary Figure 1 and Supplementary Table 1), which is consistent with previous reports (Leporé et al., 2010; Modi et al., 2012; Pan et al., 2007; Ptito et al., 2008). These regions included the bilateral V1, in which activity showed weaker functional coupling with the SMA seed region in the blind group compared to that in the sighted group (Table 6 and Figure 4a in the text). We also found a reduction of GM volume in the thalamus and cerebellar hemisphere (Lobule Crus I) in the right hemisphere.

Recently, it was shown in sighted people that GM volume expands in early sensory regions in association with individual preference in sensory processing (Yoshimura et al., 2017). The superior temporal region expanded in the present blind group corresponded well to the region reported to be expanded in sighted people with preference for auditory processing. Likewise, the precentral rolandic region expanded in the present blind group also fit well with the region expanded in sighted people with preference for tactile processing in their daily lives (Yoshimura et al., 2017). The results indicate that compared to sighted participants, blind participants may rely more on auditory and tactile processing in general, most likely to compensate for the lack of visual processing in their daily lives, which was indicated by the GM volume atrophy in the blind participants. This view seems to be compatible with the superior ability for auditory source localization in blind people (Röder et al., 1999) and superior haptic-based mental rotation ability (Carpenter & Eisenberg, 1978) especially in congenitally blind people who can read Braille.

The present expansion of the hippocampal region is also consistent with previous reports (Fortin et al., 2008; Leporé et al., 2009). This could be related to the fact that blind people have to memorize extensive information about their living environment in order to compensate for their lack of immediate updating of spatial information normally performed by the visual system. Greater hippocampal volume could be associated with better ability to remember routes in a novel maze environment (Fortin et al., 2008).

Finally, knowledge is still limited on the expansion of bilateral medial frontal regions. However, these regions seem to encompass those associated with self-referential (reflective) processing (Amodio & Frith, 2006; Decety & Sommerville, 2003; Macrae et al., 2004; Northoff et al., 2006; Ochsner et al., 2005; Vogeley & Fink, 2003). Thus, we speculate that the medial frontal expansion may reflect a more self-focused information processing style in blind people, rather than massive information processing about the external world typically captured by the visual system.

(Supplementary Figure 1 and Supplementary Table 1 around here)


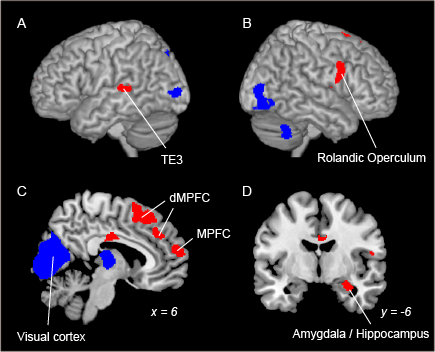


Supplementary Figure 1

Brain regions (red sections) in which GM volume expanded in the blind group compared to that in the sighted group, and those (blue sections) in which GM volume was reduced in the blind group. The regions are superimposed on the MNI standard anatomical image. A: left hemisphere, B: right hemisphere, C: sagittal section of x = +6, D: coronal section of y = -6. Detailed anatomical information is summarized in Supplementary Table 1. MPFC: medial prefrontal cortex.

***Supplementary Table 1***

**References**

Amodio, D. M., & Frith, C. D. (2006). Meeting of minds: the medial frontal cortex and social cognition. [Review Article]. *Nature Reviews Neuroscience, 7*, 268, doi: https://doi.org/10.1038/nrn1884.

Ashburner, J. (2007). A fast diffeomorphic image registration algorithm. *NeuroImage, 38*(1), 95-113, doi: https://doi.org/10.1016/j.neuroimage.2007.07.007.

Ashburner, J. (2010). VBM tutorial. *Tech. repWellcome Trust Centre for Neuroimaging, London, UK*.

Carpenter, P. A., & Eisenberg, P. (1978). Mental rotation and the frame of reference in blind and sighted individuals. *Perception & Psychophysics, 23*(2), 117-124, doi: https://doi.org/10.3758/BF03208291.

Decety, J., & Sommerville, J. A. (2003). Shared representations between self and other: a social cognitive neuroscience view. *Trends in Cognitive Sciences, 7*(12), 527-533, doi:https://doi.org/10.1016/j.tics.2003.10.004.

Fortin, M., Voss, P., Lord, C., Lassonde, M., Pruessner, J., Saint-Amour, D., et al. (2008). Wayfinding in the blind: larger hippocampal volume and supranormal spatial navigation. *Brain, 131*(11), 2995-3005, doi: https://doi.org/10.1093/brain/awn250.

Friston, K. J., Worsley, K. J., Frackowiak, R. S. J., Mazziotta, J. C., & Evans, A. C. (1994). Assessing the significance of focal activations using their spatial extent. *Human Brain Mapping, 1*(3), 210-220, doi: https://doi.org/10.1002/hbm.460010306.

Guillot, A., Collet, C., Nguyen, V. A., Malouin, F., Richards, C., & Doyon, J. (2009). Brain activity during visual versus kinesthetic imagery: An fMRI study. *Human Brain Mapping, 30*(7), 2157-2172, doi: https://doi.org/10.1002/hbm.20658.

Leporé, N., Shi, Y., Lepore, F., Fortin, M., Voss, P., Chou, Y.-Y., et al. (2009). Pattern of hippocampal shape and volume differences in blind subjects. *NeuroImage, 46*(4), 949-957, doi: https://doi.org/10.1016/j.neuroimage.2009.01.071.

Leporé, N., Voss, P., Lepore, F., Chou, Y.-Y., Fortin, M., Gougoux, F., et al. (2010). Brain structure changes visualized in early- and late-onset blind subjects. *NeuroImage, 49*(1), 134-140, doi: https://doi.org/10.1016/j.neuroimage.2009.07.048.

Macrae, C. N., Moran, J. M., Heatherton, T. F., Banfield, J. F., & Kelley, W. M. (2004). Medial Prefrontal Activity Predicts Memory for Self. *Cerebral Cortex, 14*(6), 647-654, doi: https://doi.org/10.1093/cercor/bhh025.

Modi, S., Bhattacharya, M., Singh, N., Tripathi, R. P., & Khushu, S. (2012). Effect of visual experience on structural organization of the human brain: A voxel based morphometric study using DARTEL. *European Journal of Radiology, 81*(10), 2811-2819, doi: https://doi.org/10.1016/j.ejrad.2011.10.022.

Naito, E. (1994). Controllability of notor imagery and transformation of visual imagery. *Perceptual and Motor Skills, 78*(2), 479-487, doi: https://doi.org/10.2466/pms.1994.78.2.479.

Naito, E., Kochiyama, T., Kitada, R., Nakamura, S., Matsumura, M., Yonekura, Y., et al. (2002). Internally simulated movement sensations during motor imagery activate cortical motor areas and the cerebellum. *The Journal of Neuroscience, 22*(9), 3683-3691.

Nishida, T., Katube, A., Inomata, K., Okazawa, Y., Ito, M., Kayama, S., et al. (1986). A new test for controllability of motor imagery: the examination of its validity and reliability. *Japanese Journal of Physical Education, 31*(1), 13-22.

Northoff, G., Heinzel, A., de Greck, M., Bermpohl, F., Dobrowolny, H., & Panksepp, J. (2006). Self-referential processing in our brain—A meta-analysis of imaging studies on the self. *NeuroImage, 31*(1), 440-457, doi: https://doi.org/10.1016/j.neuroimage.2005.12.002.

Ochsner, K. N., Beer, J. S., Robertson, E. R., Cooper, J. C., Gabrieli, J. D. E., Kihsltrom, J. F., et al. (2005). The neural correlates of direct and reflected self-knowledge. *NeuroImage, 28*(4), 797-814, doi: https://doi.org/10.1016/j.neuroimage.2005.06.069.

Pan, W. J., Wu, G., Li, C. X., Lin, F., Sun, J., & Lei, H. (2007). Progressive atrophy in the optic pathway and visual cortex of early blind Chinese adults: A voxel-based morphometry magnetic resonance imaging study. *NeuroImage, 37*(1), 212-220, doi: https://doi.org/10.1016/j.neuroimage.2007.05.014.

Ptito, M., Schneider, F. C. G., Paulson, O. B., & Kupers, R. (2008). Alterations of the visual pathways in congenital blindness. [journal article]. *Experimental Brain Research, 187*(1), 41-49, doi: https://doi.org/10.1007/s00221-008-1273-4.

Röder, B., Teder-Sälejärvi, W., Sterr, A., Rösler, F., Hillyard, S. A., & Neville, H. J. (1999). Improved auditory spatial tuning in blind humans. *Nature, 400*, 162, doi: https://doi.org/10.1038/22106.

Ridgway, G. R., Omar, R., Ourselin, S., Hill, D. L. G., Warren, J. D., & Fox, N. C. (2009). Issues with threshold masking in voxel-based morphometry of atrophied brains. *NeuroImage, 44*(1), 99-111, doi: https://doi.org/10.1016/j.neuroimage.2008.08.045.

Vogeley, K., & Fink, G. R. (2003). Neural correlates of the first-person-perspective. *Trends in Cognitive Sciences, 7*(1), 38-42, doi: http://dx.doi.org/10.1016/S1364-6613(02)00003-7.

Yoshimura, S., Sato, W., Kochiyama, T., Uono, S., Sawada, R., Kubota, Y., et al. (2017). Gray matter volumes of early sensory regions are associated with individual differences in sensory processing. *Human Brain Mapping, 38*(12), 6206-6217, doi: https://doi.org/10.1002/hbm.23822.

Zatorre, R. J., Fields, R. D., & Johansen-Berg, H. (2012). Plasticity in gray and white: neuroimaging changes in brain structure during learning. *Nature Neuroscience, 15*(4), 528-536, doi: https://doi.org/10.1038/nn.3045.
